# Discussion and Analysis of Dumbbell Defect-Ground-Structure (DB-DGS) Resonators for Sensing Applications from a Circuit Theory Perspective

**DOI:** 10.3390/s21248334

**Published:** 2021-12-13

**Authors:** Lijuan Su, Paris Vélez, Jonathan Muñoz-Enano, Ferran Martín

**Affiliations:** CIMITEC, Departament d’Enginyeria Electrònica, Universitat Autònoma de Barcelona, 08193 Bellaterra, Spain; Lijuan.Su@uab.cat (L.S.); Paris.Velez@uab.cat (P.V.); Jonatan.Munoz@uab.cat (J.M.-E.)

**Keywords:** slot resonator, dumbbell defect-ground structure (DB-DGS), microwave sensor, parameter extraction, microstrip technology

## Abstract

Microstrip transmission lines loaded with dumbbell defect-ground-structure (DB-DGS) resonators transversally oriented have been exhaustively used in microwave circuits and sensors. Typically, these structures have been modelled by means of a parallel LC resonant tank series connected to the host line. However, the inductance and capacitance of such model do not have a physical meaning, since this model is inferred by transformation of a more realistic model, where the DB-DGS resonator, described by means of a resonant tank with inductance and capacitance related to the geometry of the DB-DGS, is magnetically coupled to the host line. From parameter extraction, the circuit parameters of both models are obtained by considering the DB-DGS covered with semi-infinite materials with different dielectric constant. The extracted parameters are coherent and reveal that the general assumption of considering the simple LC resonant tank series-connected to the line to describe the DB-DGS-loaded line is reasonable with some caution. The implications on the sensitivity, when the structure is devoted to operating as a permittivity sensor, are discussed.

## 1. Introduction

**Figure 1:**
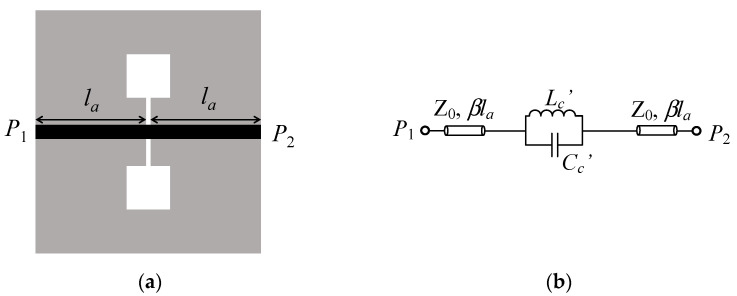
Typical topology (**a**) and most commonly accepted circuit model (**b**) of a microstrip transmission line loaded with a transversally oriented DB-DGS resonator, etched in the ground plane. *L_c_*′ and *C_c_*′ are related to the inductance and capacitance, respectively, of the DB-DGS resonator, whereas the transmission line (in black color) is described by the pair of transmission line sections with characteristic impedance *Z*_0_, length *l_a_*, and phase constant *β*. The ground plane is depicted in grey color. Losses are neglected in this model.

The dumbbell defect-ground-structure (DB-DGS) resonator [1,2] is a slot resonator exhaustively used in microwave engineering for the implementation of circuits (mainly filters [3,4,5,6,7,8,9]) and sensors [10,11,12,13]. DB-DGS resonators are typically combined with microstrip transmission lines. The typical topology of a DB-DGS-loaded microstrip line is depicted in Figure 1, where the resonant element is transversally etched in the ground plane. This structure exhibits a notched response with a transmission zero at the fundamental resonance frequency of the DB-DGS resonator, and the structure has been typically modelled by means of a parallel resonant tank series-connected to the host transmission line [1,2] (see Figure 1b).

The notched response of DB-DGS-loaded microstrip lines can be exploited for the implementation of notch and stopband filters [3,4,5,6], as well as for the design of common-mode filters in differential lines [7,9]. However, slot resonators, including the DB-DGS resonator, are also of interest for the implementation of planar microwave sensors devoted to permittivity measurements, and to the characterization of materials (including solids and liquids) [10,11,12,13]. The reason is that the resonance frequency of these slot resonators is very sensitive to the dielectric properties of the material surrounding the resonator (in contact or in close proximity to it). Thus, the canonical output variable in these permittivity sensors is the resonance frequency of the resonant element (and eventually the notch magnitude) very sensitive to the permittivity of the so-called material under test (MUT).

For both circuit and sensor design, accurate circuit models of the structures under consideration (able to link the geometry parameters to the lumped elements of the model) are very convenient. As mentioned, the most accepted circuit model of a DB-DGS-loaded microstrip line is the one depicted in Figure 1b, where the DB-DGS resonator is modelled by means of the parallel resonant tank, series connected to the line. It has been argued that this model accounts for the return current path in the ground plane, either across the narrow slot of the DB-DGS resonator (providing the capacitive effect), or surrounding the apertures (inductive effect) [1,2,9]. Thus, according to this physical interpretation, the parallel combination of the inductance and the capacitance of the DB-DGS, as well as the connection of this parallel LC tank in series with the line, is clear. However, the fact that the resonator is etched in the ground plane generates some doubts concerning the validity of the above-mentioned physical interpretation of the model parameters. Indeed, in microstrip-to-slot-line transitions, it has been reported that the excitation of the slot line is due to magnetic coupling [14,15]. In the structure of Figure 1, rather than a microstrip-to-slot-line transition, a microstrip line excites a slot resonator with apertures in the extremes, i.e., a DB-DGS resonator. However, it is apparent that the driving mechanism should be the same. Indeed, in previous papers [16,17], a different model of the DB-DGS-loaded microstrip line, which accounts for the magnetic coupling between the host line and the resonant element, has been reported. Actually, the simple model of Figure 1 can be derived from such model by element transformation. However, the interpretation of the elements of the parallel resonant tank of Figure 1, as those describing the capacitance and inductance of the DB-DGS resonator, does not seem to be correct or, at least, accurate. In this paper, all these aspects are discussed in detail, and the implications on the functionality of the DB-DGS-loaded line as permittivity sensor are analyzed.

## 2. Model and Analysis

Any planar microwave resonator, including both metallic and slot-based resonators, can be modelled by means of a LC resonant tank in the vicinity of the fundamental resonance. Additionally, if the considered resonator is electrically small, then the LC resonant tank provides a good description of the resonant element in a relatively broad band that extends significantly above the fundamental resonance. The DB-DGS resonator is the complementary, or dual, counterpart of the so-called step-impedance-resonator (SIR) [18,19]. Thus, likewise the SIR, the DB-DGS resonator can be considered an electrically small resonator, by virtue of the high contrast between the width of the narrow (central) slot and the apertures at the extremes. Whereas in the SIR the inductance is mainly due to the narrow strip and the capacitance to the metallic patches at the extremes [18,19], it follows from duality considerations [20,21,22] that the narrow slot of the DB-DGS resonator determines its capacitance, whilst the inductance is mainly determined by the size of the apertures. However, in the model depicted in Figure 1b, the reactive element values *L_c_*′ and *C_c_*′ are not the inductance and capacitance, respectively, of the DB-DGS resonator. Nevertheless, as it will be shown later, *L_c_*′ and *C_c_*′ are related to the inductance *L_c_* and capacitance *C_c_*, respectively, of the DB-DGS resonator.

**Figure 2:**
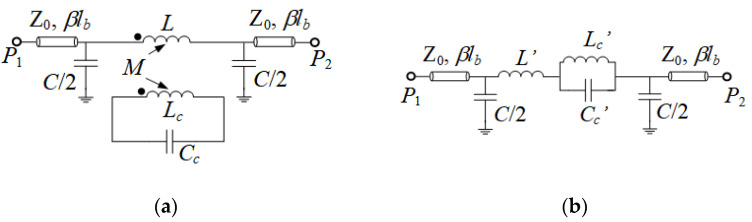
Circuit model of the DB-DGS-loaded microstrip line with the resonator magnetically coupled to the line (**a**) and transformed model (**b**).

At the fundamental resonance frequency, a DB-DGS resonator exhibits a magnetic wall at its symmetry plane (the axis of the microstrip line in Figure 1a). Consequently, the fundamental resonance of the DB-DGS resonator can be driven by means of a time-varying magnetic field with a non-negligible component in the plane of the particle, in the direction orthogonal to the magnetic wall. It is obvious that the magnetic field generated by the line satisfies such requirement. Therefore, the line present on top of the DB-DGS can magnetically excite the resonant element. Thus, a more realistic circuit model of the DB-DGS-loaded microstrip transmission line is the one depicted in Figure 2a [16,17]. In this model, *L_c_* and *C_c_* describe the DB-DGS resonator, as indicated before. Particularly, the capacitance *C_c_* is determined by the length and width of the narrow (central) slot, whereas the size of the apertures is the main parameter determining the inductance value *L_c_*. In order to describe the magnetic coupling between the microstrip line and the resonator, we should model a portion of the line (with length *l*, where *l* < *l_a_*) by means of its inductance *L* and capacitance *C*. By this means, we can define a mutual inductance *M*, describing the magnetic coupling between the line and the DB-DGS resonator. In coherence with Figure 1, the length of the transmission line sections in Figure 2 is simply *l_b_* = *l_a_* − *l*/2.

The series branch in the circuit of Figure 2a can be transformed to a parallel resonator series-connected to an inductor (Figure 2b). The elements of the series branch in the circuit of Figure 2b are related to the elements of the original circuit of Figure 2a through the following equations [17,22]

(1) $$L^{\prime }=L-{L^{\prime }}_{c}$$

(2) $${L_{c}}^{\prime }=C_{c}M^{2}\omega_{0}^{2}$$

(3) $${C_{c}}^{\prime }=\frac{L_{c}}{M^{2}\omega_{0}^{2}}$$

where $\omega_{0}^{2}=1/L_{c}C_{c}$ = $1/{L_{c}}^{\prime }{C_{c}}^{\prime }$ ($\omega_{0}$ being the angular frequency at the fundamental resonance of the DB-DGS resonator). If we compare the circuit of Figure 2b with the one in Figure 1b, the difference is the presence of the inductance *L*′ and the capacitance *C* in the circuit of Figure 2b, not present in the circuit of Figure 1b. However, as it will be later demonstrated, *L*′ and *C* describe the transmission line section of length *l*. Such line section, plus the lines of length *l_b_* in the circuit of Figure 2b, give the total line length *l_a_* of the circuit of Figure 1b.

According to the preceding paragraph, the circuits of Figure 1b and Figure 2b or Figure 2a, are effectively equivalent, and, therefore, it is licit to consider valid the circuit of Figure 1b. However, in the circuit of Figure 1b, the reactive elements *L_c_*′ and *C_c_*′ do not correspond to the inductance and capacitance of the DB-DGS resonator, as anticipated before. Nevertheless, the reactive elements of the resonators in both models (Figure 2a,b) are intimately related. By introducing $\omega_{0}^{2}=1/L_{c}C_{c}$ in Equations (2) and (3) one obtains:

(4) $${L_{c}}^{\prime }=\frac{M^{2}}{L_{c}}$$

(5) $${C_{c}}^{\prime }=\frac{L_{c}^{2}}{M^{2}}C_{c}$$

providing the relationship between the resonator elements in both models. Note, however, that there is a fundamental difference between the models of Figure 2a,b. Whereas the series branch in the model of Figure 2a depends on four reactive parameters (*L*, *M*, *L_c_* and *C_c_*), the series branch in the circuit model of Figure 2b, equivalent to Figure 1b, contains only three reactive elements (*L*′, *L_c_*′ and *C_c_*′). Thus, from the information of the reactive parameters of the circuit of Figure 2b, easily obtained from parameter extraction [22,23,24], it is not possible to obtain *M*, *L_c_* and *C_c_*. By contrast, *L* can be easily isolated from Equation (1). In order to independently obtain *M*, *L_c_* and *C_c_* from *L_c_*′ and *C_c_*′, at least one of the three reactive parameters *M*, *L_c_* or *C_c_* must be determined from an independent method. Let us discuss this aspect in the next section, where the implications of the transformation Equations (1)–(3) or Equations (4) and (5), on the functionality of the DB-DGS-loaded line as permittivity sensor are discussed.

## 3. Parametric Analysis and Implications for Permittivity Sensing

**Figure 3:**
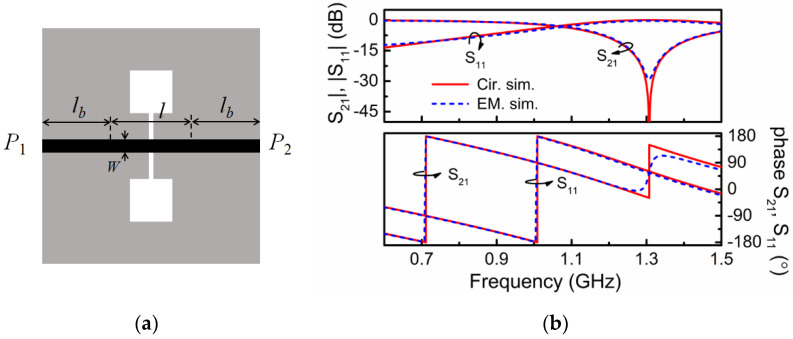
Layout (**a**) and frequency response (**b**) of the considered DB-DGS. DB-DGS dimensions are: length of the narrow slot 2*l* = 24 mm, width of the slot 0.2 mm, side length of the square apertures 12 mm. The electromagnetic simulations are lossless.

Let us consider as case study a microstrip transmission line loaded with a DB-DGS resonator with the dimensions depicted in the caption of Figure 3, where the specific topology is depicted. The width of the line (*W* = 1.1 mm) provides a 50-Ω characteristic impedance with the considered substrate parameters (dielectric constant *ε_r_* = 10.2 and thickness *h* = 1.27 mm). The response of this structure inferred from electromagnetic simulation is depicted in Figure 3b. From this response, we have extracted the parameters of the circuit of Figure 2b, i.e., *L*′ = 5.12 nH, *L_c_*′ = 4.10 nH, *C_c_*′ = 3.63 pF and *C* = 2.21 pF (the parameter extraction method reported in [22,23,24] has been used). Actually, such parameters correspond to the DB-DGS coupled to a line section of length *l* = 12 mm (note that the obtained values of *L*′ and *C* are compatible with a 50-Ω characteristic impedance line of the considered length, i.e., 12 mm). With such parameters, the circuit response is found to be roughly undistinguishable from the electromagnetic response in the considered frequency range. However, as mentioned, it is not possible to infer *M*, *L_c_* and *C_c_* by means of Equations (1)–(3), or Equations (4) and (5). In the circuit of Figure 2a, only the capacitance *C*, invariable as compared to the one of Figure 2b, and *L* (= *L*′+ *L_c_*′ = 9.21 nH), can be univocally determined.

**Figure 4:**
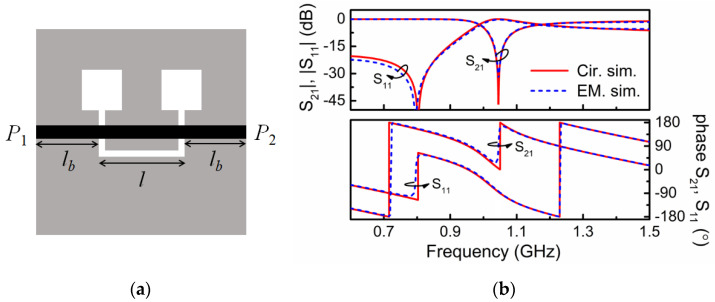
Layout (**a**) and lossless frequency response (**b**) of the folded and 90° rotated DB-DGS. Dimensions are identical to those of Figure 3.

**Figure 5:**
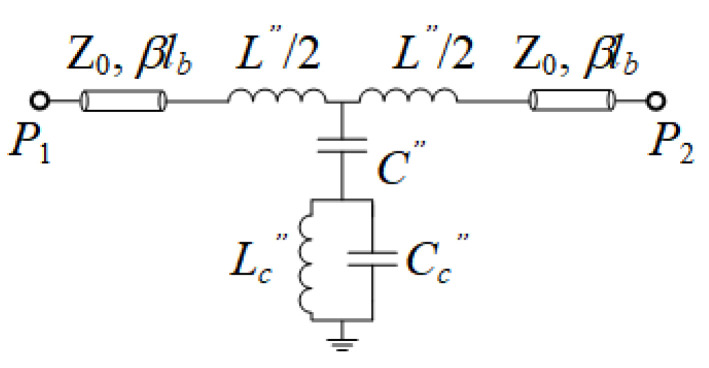
Circuit model of the microstrip line loaded with the folded and rotated (90°) DB-DGS resonator.

In order to be able to obtain the circuit parameters of the original model of Figure 2a, we have considered an auxiliary structure, i.e., a microstrip line loaded with an identical DB-DGS resonator, but folded and rotated 90°, as depicted in Figure 4 (this structure can also be useful for permittivity sensing). In this structure, the magnetic coupling cancels because the symmetry plane of the particle, a magnetic wall, is orthogonally oriented with regard to the line axis. However, there is an electric dipole in the vertical direction, since the resonator is folded [17]. Therefore, with such orientation and folding, the DB-DGS resonator is excited exclusively by the electric field generated by the line, with a significant component in the vertical direction. The circuit model for this structure is thus the one shown in Figure 5. The parameters of this model can be easily extracted (the method reported in [23] has been applied), and the results are as follows: *L*″ = 9.27 nH, *C*″ = 2.07 pF, *C_c_*″ = 4.23 pF and *L_c_*″ = 3.68 nH (the electromagnetically simulated and circuit response are depicted in Figure 4b). As it can be appreciated, *L*″ ≈ *L* and *C*″ ≈ *C*, i.e., the parameters describing the line section of length *l* do not appreciable change by rotating and folding the DB-DGS resonator. In this paper, we do assume that the capacitance *C_c_* of the unfolded DB-DGS does not change as compared to the one of the folded particles, *C_c_*″. This latter hypothesis is reasonable, since the capacitance of the resonator mainly depends on the length and width of the narrow (central) slot and it is not expected that such capacitance varies significantly by rotating and folding the DB-DGS. By considering that *C_c_*″ = *C_c_*, the remaining parameters of the circuit of Figure 2a, i.e., *M* and *L_c_*, can be perfectly determined from inversion of Equations (4) and (5), or Equations (1)–(3), and the results are *M* = 3.80 nH and *L_c_* = 3.52 nH. Let us clarify that in the circuit of Figure 5, the capacitance *C_c_*″ can be univocally determined from parameter extraction. However, from the extracted parameters of the circuit of Figure 2b, it is not possible through inversion of Equations (4) and (5) or Equations (1)–(3), to univocally determining the elements of the series branch in the circuit of Figure 2a, particularly *M*, *L_c_* and *C_c_*. It is neither possible to infer these elements from direct parameter extraction, since, given a response, for example the one of Figure 3b, corresponding to the structure of Figure 3a, there are many combinations of the triad *M*, *L_c_* and *C_c_* providing such response, as inspection of Equations (1)–(3) and Equations (4) and (5) reveals.

Note that the inductance of the rotated and folded resonator varies slightly, as compared to the one of the unfolded and transversally oriented DB-DGS, i.e., *L_c_*″ ≠ *L_c_*. This is attributed to the fact that the current path from the inner to the outer metallic region of the folded particle is very different from the inductive current path in the unfolded resonator. Moreover, the influence of the line strip on the inductance of the DB-DGS is more severe if the particle is folded and oriented as shown in Figure 4a.

To gain insight on the behavior of the reactive elements when dimensions are modified, we have considered variations in the length of the narrow slot of the DB-DGS (keeping the other dimensions unaltered). This should mainly (although not exclusively) affect the capacitance of the resonant particle, and it is reasonable to consider the effects of varying such dimension. The reason is that in the functionally of the structure under consideration as permittivity sensor, the material under test (MUT), placed on top of the DB-DGS, modifies the capacitance of the particle. Nevertheless, it should be mentioned that it is also expected that the inductance of the particle, *L_c_*, as well as the mutual inductance, *M*, experience a certain variation by varying the length of the narrow slot of the DB-DGS resonators. By contrast, such inductive elements are not altered by the presence of a dielectric material (MUT) on top of the DB-DGS.

**Figure 6:**
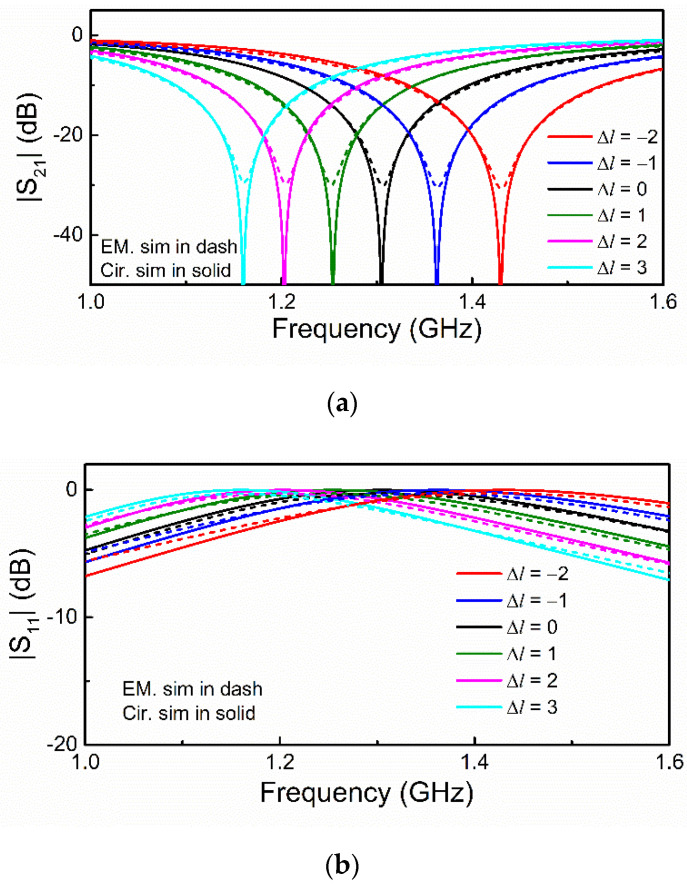
Responses (circuit and electromagnetic) of the DB-DGS-loaded microstrip line for various lengths of the narrow slot of the DB-DGS. (**a**) Magnitude of the transmission coefficient; (**b**) magnitude of the reflection coefficient. Minus sign in Δ*l* means reducing the length of the narrow slot.

Figure 6 depicts the electromagnetically simulated responses that are obtained by varying the slot length in a quantity Δ*l* as compared to the nominal value. As expected, the resonance frequency decreases with the slot length. The extracted parameters of the circuit of Figure 2b are shown in Table 1. The circuit responses inferred from the extracted parameters for each case are also depicted in Figure 6 for comparison purposes, and the agreement is good in all the cases. By applying the previous procedure, the parameters of the model of Figure 2a have been obtained, and the results are also depicted in Table 1. The capacitance of the resonator, *C_c_*, and the transformed capacitance, *C_c_*′, both increase with the slot length, as expected, but it can be seen that *L_c_*, *L_c_*′ and *M* experience an appreciable variation, as well. The important aspect of this analysis is that *C_c_* and *C_c_*′ increase with the slot length. However, the capacitance that has a physical interpretation, i.e., the one of the DB-DGS resonator (attributed to the narrow slot), is *C_c_*, rather than *C_c_*′.

**Figure 7:**
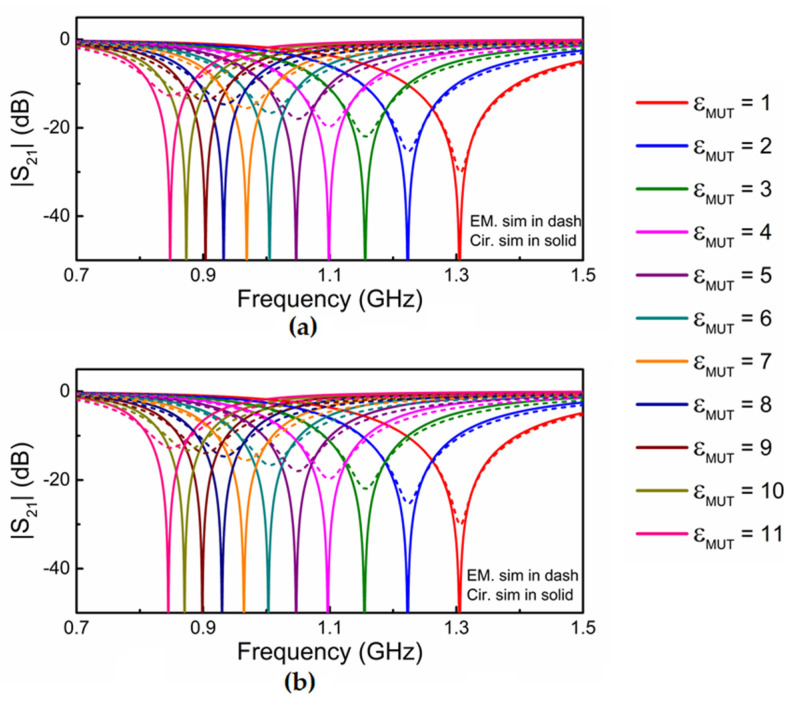
Responses (circuit and electromagnetic) of the DB-DGS-loaded microstrip line for various dielectric constants of the MUT. (**a**) Circuit responses obtained with *C_c_* inferred by curve fitting; (**b**) circuit responses obtained with *C_c_* calculated from (6).

Let us next consider the effects of varying the capacitance of the DB-DGS resonator, but rather than by tailoring particle dimensions (narrow slot length), by covering the DB-DGS by means of a dielectric MUT with semi-infinite length in the vertical direction, and with lateral dimensions extending far beyond the boundaries of the DB-DGS (semi-infinite approximation). This approximation, which means that the electric field generated by the DB-DGS does not reach the limits of the MUT, is not a sensor requirement. However, it eases the analysis, to be carried out later, and does not represent a loss of generality. The responses of the DB-DGS-loaded line with nominal dimensions (i.e., with narrow slot length 2*l* = 24 mm) for MUTs with various dielectric constant are depicted in Figure 7a. By covering the DB-DGS with a dielectric material, *C_c_* should change, but not *L_c_* and *M*, as indicated before. Consequently, according to Equations (4) and (5), *C_c_*′ should vary with the dielectric constant of the MUT, but not *L_c_*′. If we infer the circuit responses for the different dielectric constant of the MUT using the different values of *C_c_*′ that are required to adjust the resonance frequency to the simulated value (keeping the other parameters invariable), the agreement with the simulations is very good. The values of *C_c_*′ and *C_c_* for the different dielectric constants are shown in Table 2. Let us call *C_c,air_* the value of *C_c_* corresponding to the DB-DGS surrounded by air (with dielectric constant *ε_MUT_* = 1). For a semi-infinite MUT with arbitrary dielectric constant, the capacitance of the resonator should vary according to [25,26]

(6) $$C_{c}=C_{c,air}\frac{\varepsilon_{r,eq}+\varepsilon_{MUT}}{\varepsilon_{r,eq}+1}$$

where $\varepsilon_{r,eq}$ is the so-called equivalent dielectric constant of the substrate, defined in [26], which takes into account the finite thickness of the substrate. Namely, if the substrate satisfies the semi-infinite approximation, the capacitance *C_c_* is given by (6) with $\varepsilon_{r,eq}$ replaced with $\varepsilon_{r}$, the substrate dielectric constant [25,26]. However, if the field lines generated in the slot of the DB-DGS resonator reach the opposite substrate interface, then the capacitance should be calculated according to (6). Equation (6) assumes that the capacitance of the resonator *C_c_* is given by the parallel combination of the capacitances of the upper (MUT) and lower (substrate) subspaces of the slot, a hypothesis that is valid provided there is a quasi-magnetic wall in the plane of the DB-DGS resonator. In [26], it was demonstrated that for any reasonable combination of substrate thickness, substrate dielectric constant, and slot width, it can be considered that the plane of the particle is a magnetic wall to a very good approximation, thereby pointing out the validity of (6). The physical interpretation of $\varepsilon_{r,eq}$ is clear, namely, it is the value of the dielectric constant that a hypothetical semi-infinite substrate should exhibit in order to provide a contribution to the capacitance of the particle identical to the one of the actual (finite) substrate. The method for the calculation of $\varepsilon_{r,eq}$ is simple. Let us call *f*_0,air_ and *f*_0,MUT_ the resonance frequencies of the DB-DGS resonator when it is unloaded and loaded with a certain (semi-infinite) MUT, respectively. The ratio of these frequencies is

(7) $$\frac{f_{0,air}}{f_{0,MUT}}=\sqrt{\frac{\varepsilon_{r,eq}+\varepsilon_{MUT}}{\varepsilon_{r,eq}+1}}$$

Provided the inductance of the DB-DGS resonator does not vary with the presence of the MUT. Since these frequencies can be easily obtained from electromagnetic simulation, it follows that $\varepsilon_{r,eq}$ can be inferred from (7). The calculated value is $\varepsilon_{r,eq}$ = 6.30. Obviously,$\;\varepsilon_{r,eq}$ does not depend on the MUT. It only depends on the characteristics of the substrate and slot width. Thus, the value of $\varepsilon_{r,eq}$ should be the same, regardless of the considered value of the dielectric constant of the MUT, $\varepsilon_{MUT}$. This aspect has been corroborated by considered different $\varepsilon_{MUT}$ values, providing roughly identical values of $\varepsilon_{r,eq}$ (not shown). Indeed, the value of $\varepsilon_{r,eq}$ given above is an average value, but the standard deviation is very small.

The capacitance values given by (6) are also included in Table 2 and are very close to the values of *C_c_* inferred by inversion of (4–5) using the values of *C_c_*′ adjusted to fit the resonance values of the different MUTs. The circuit responses with the values of *C_c_* calculated by means of (6) are compared to the simulated responses in Figure 7b, and the agreement is also very good.

From (4–5), it follows that *C_c_*′ should exhibit a dependence with *ε_MUT_* given by

(8) $${C_{c}}^{\prime }=\frac{L_{c}^{2}}{M^{2}}C_{c,air}\frac{\varepsilon_{r,eq}+\varepsilon_{MUT}}{\varepsilon_{r,eq}+1}$$

Thus, if we call ${{C_{c}}^{\prime }}_{air}=C_{c,air}L_{c}^{2}/M^{2}$, it is clear that the capacitance of the model of Figure 2b has a dependence on *ε_MUT_* identical to the one of the capacitance of the circuit of Figure 2a, the actual capacitance of the DB-DGS resonator. Moreover, the resonance frequency is identical in both models. Therefore, it is obvious that the sensitivity does not depend on whether the circuit model of Figure 2b or the more realistic model of Figure 2a is considered.

To gain more insight on this latter aspect, let us calculate the sensitivity, namely, the variation experienced by the output variable, the resonance frequency, with the input variable, the dielectric constant of the MUT, i.e.,

(9) $$S=\frac{df_{0}}{d\varepsilon_{MUT}}$$

If the model of Figure 2b is considered, the previous expression can be decomposed in two terms as follows

(10) $$S=\frac{df_{0}}{d{C_{c}}^{\prime }}\frac{d{C_{c}}^{\prime }}{d\varepsilon_{MUT}}$$

where the first and second terms are given by

(11) $$\frac{df_{0}}{d{C_{c}}^{\prime }}=-\frac{1}{2}\cdot \frac{f_{0}}{{C_{c}}^{\prime }}$$

and

(12) $$\frac{d{C_{c}}^{\prime }}{d\varepsilon_{MUT}}=\frac{{{C_{c}}^{\prime }}_{air}}{\varepsilon_{r}+1}$$

respectively. Introducing (11) and (12) in (10), and using (8), the sensitivity is found to be

(13) $$S=-\frac{1}{2}\cdot \frac{f_{0}}{\varepsilon_{r,eq}+\varepsilon_{MUT}}$$

Alternatively, if the model of Figure 2a is the considered one, the sensitivity is also given by (10), with *C_c_*′ replaced with *C_c_*. Equations (11) and (12) are also valid with *C_c_*′ replaced with *C_c_*, and $C_{c}\prime_{air}$ replaced with $C_{c,air}$. Note, however, that the resulting sensitivity is also given by (13), since the resonance frequency is identical in both models (see Equations (1)–(3). Therefore, according to this analysis, it can be unequivocally concluded that despite the fact that the capacitance *C_c_*′ does not correspond to the capacitance of the DB-DGS resonator, using the model of Figure 2b for design purposes, as usually done with an eye towards simplification, is perfectly licit.

**Figure 8:**
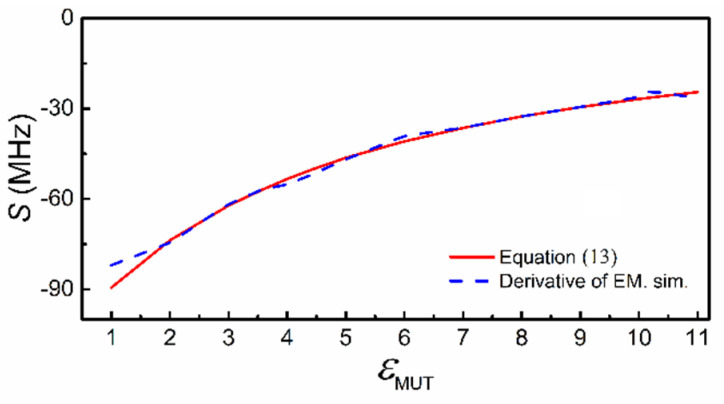
Sensitivity inferred from theory and from electromagnetic simulation.

**Figure 9:**
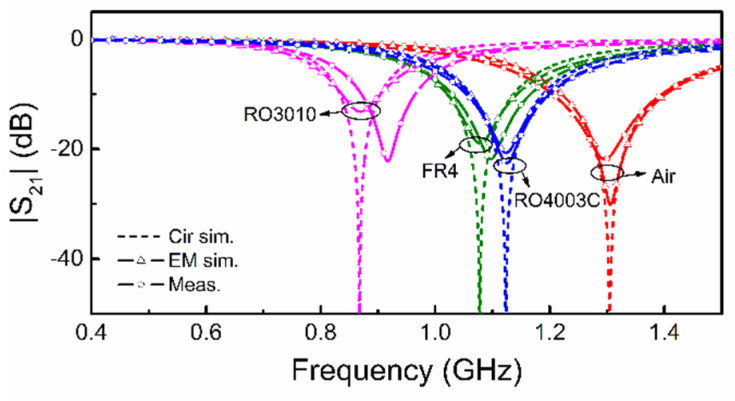
Experimental validation. The dielectric constant of the considered samples is 10.2 (*Rogers RO3010*), 4.4 (FR4), 3.55 (*Rogers RO4003C*) and 1 (air), with 4 pieces of the slabs stacked together (around 4 mm) for each type of samples.

The sensitivity given by (13) is compared with the sensitivity inferred from the simulated data (resonances) of Figure 7, and the agreement is very good, as shown in Figure 8. This points out the validity of the previous sensitivity analysis. Note, however, that inferring the sensitivity from experimental results is difficult due to the lack of data points. Namely, the number of potential samples with different dielectric constant is limited. Nevertheless, we have experimentally validated the reported circuit models by covering the sensor structure of Figure 3 with various semi-infinite materials, particularly uncladded substrates available in our laboratory. Figure 9 depicts the response for such materials (indicated), and the agreement between experimental, circuit and electromagnetic simulation is good. Small discrepancies are due to fabrication related tolerances, and to the fact that the samples are actually stacked slabs, with certain air gap effect between them (there are not thick enough substrates so as to consider them semi-infinite).

## 4. Conclusions

In conclusion, in this paper we have pointed out that the generalized assumption that the circuit model of a microstrip line loaded with a dumbbell defect-ground-structure (DB-DGS) resonator transversally etched in the ground plane is a simple parallel resonant tank series-connected to the line is correct, but with some considerations. Specifically, the reactive elements of such simple model do not actually correspond to the inductance and capacitance of the DB-DGS resonator. Such reactive elements are the result of a circuit transformation from a more realistic model, where the DB-DGS resonator is magnetically coupled to the host microstrip line. However, the analysis carried out in this paper reveals that, from a practical viewpoint, the simplified model can be used not only for circuit design purposes, but also for the prediction of the sensitivity, a relevant performance parameter in applications of the considered DB-DGS-based structure to dielectric constant sensing.

## Figures and Tables

**Table 1:** Circuit parameters of the circuits of Figure 2 for different lengths of the narrow slot of the DB-DGS resonator.

Δ*l* (mm)	*L*′/*L* (nH)	*L_c_*′/*L_c_* (nH)	*C_c_*′/*C_c_* (pF)	*M* (nH)
−2	4.96/8.73	3.77/3.15	3.28/3.93	3.45
−1	5.06/9.02	3.96/3.34	3.44/4.08	3.64
0	5.12/9.21	4.10/3.52	3.63/4.23	3.80
1	5.26/9.54	4.27/3.67	3.77/4.39	3.96
2	5.18/9.54	4.36/3.87	4.01/4.52	4.11
3	5.29/9.80	4.51/4.02	4.17/4.69	4.26

**Table 2:** Circuit parameters for different values of the dielectric constant of the MUT.

*ε_MUT_*	*C_c_*′ (pF)	*C_c_* (pF)	*C_c_* (pF) [From (6)]
1	3.63	4.23	4.22
2	4.14	4.81	4.81
3	4.63	5.39	5.39
4	5.13	5.97	5.97
5	5.65	6.57	6.55
6	6.13	7.13	7.13
7	6.59	7.67	7.71
8	7.12	8.29	8.29
9	7.58	8.82	8.87
10	8.11	9.44	9.45
11	8.61	10.02	10.02

## Data Availability

Not applicable.
